# 巨大纵隔原始神经外胚瘤1例并文献复习

**DOI:** 10.3779/j.issn.1009-3419.2013.05.10

**Published:** 2013-05-20

**Authors:** 治强 伍, 虹利 万, 敏 史, 炜 高, 占鹏 王, 惠萍 刘, 庆新 李

**Affiliations:** 1 730050 兰州，兰州军区兰州总医院普胸外科 Department of Thoracic Surgery, Lanzhou General Hospital, Lanzhou Command, Lanzhou 730050, China; 2 730050 兰州，兰州军区兰州总医院妇产科 Department of Gynecology and Obstitrics, Lanzhou General Hospital, Lanzhou Command, Lanzhou 730050, China; 3 730050 兰州，兰州军区兰州总医院病理科 Department of Pathology, Lanzhou General Hospital, Lanzhou Command, Lanzhou 730050, China

原始神经外胚层瘤（primitive neuroectodermal tumor, PNET）是一种少见高度恶性软组织肿瘤，好发于儿童及青少年，可发生于神经系统及全身软组织。起源于外周神经系统的被称之为外周性PNET，以躯干、四肢和中轴软组织多见。发生于纵隔的PNET较为罕见。兰州军区兰州总医院普胸外科近来收治1例儿童巨大纵隔PNET，经手术完整切除，较为罕见，现报告如下。

## 1 临床资料

患儿，男，3岁6个月，因“左胸痛11天，发现胸腔占位9天”入院。查体：气管略右偏，左胸饱满，左上肺呼吸音弱，左下肺呼吸音消失。胸片及胸部增强CT（图 1）示：左侧胸腔见一巨大软组织肿块，大小约10 cm×10 cm×8 cm，增强检查病灶可见不规则强化影，多考虑恶性肿瘤，肺母细胞瘤可能性大；左肺不张、实变，左侧少量胸腔积液；未见明确肿大淋巴结。颅脑CT平扫未见明确异常。胸腔彩超示：左侧胸腔内可见8.2 cm×8.7 cm的混合回声，边界欠清，形态欠规则，内部回声欠均匀，左侧胸腔内探及前后径1.5 cm的无回声区。心脏彩超示：心包积液（少量）；左室收缩功能正常；彩色血流未见异常。腹部彩超：肝、胆、脾、胰、肾声像图未见明显异常。血常规示：红细胞计数4.04×10^12^/L、血红蛋白86 g/L。动脉血气分析：pH值7.401、PCO_2_ 34.2 mmHg、PO_2_ 110.4 mmHg、碱剩余-3.1 mmol/L、实际碳酸氢根20.4 mmol/L、血氧饱和度95.3%。完善术前检查并予输血支持治疗后于2012年12月在全麻下行左侧开胸探查、纵隔肿瘤切除术。经左后外侧切口第五肋间入胸。术中见胸腔内有少量淡黄色胸腔积液约100 mL，纵隔巨大肿瘤约11 cm×8 cm×8 cm，有完整包膜，分叶状，来源于前纵隔，压迫上下叶肺及斜裂，从纵隔方向与上下叶粘连紧密；上下叶肺压迫性肺不张（图 2）。术中取部分组织送冰冻检查示（纵隔）恶性肿瘤。手术完整切除肿物，探查胸内无明显增大淋巴结。术后病理巨检见灰褐色结节样肿物一个，大小11 cm×7 cm×5 cm，表面见部分包膜。病理诊断为（纵隔）PNET。免疫组化：CD99（+）、GFAP（-）、S100灶性（+）、CgA（-）、Syn（-）、NeuN（+）、Nestin（+）、OCT3/4（-）、CD117（-）、Ki67 > 90%、CKp（+）、EMA（-）、LCA（-）、Vimentin（+）（图 3）。患儿术后恢复顺利，复查胸片左肺复张良好（图 4），顺利出院。术后已按期行辅助化疗两次，方案分别为长春新碱+多柔比星+环磷酰胺和长春新碱+表柔比星+环磷酰胺。术后1个月复查胸部CT示左肺门软组织肿块，考虑局部复发；术后2个月复查腹部彩超示腹腔多发肿大淋巴结，考虑远处转移。目前患者一般状况尚好，正治疗观察中。

**1:**
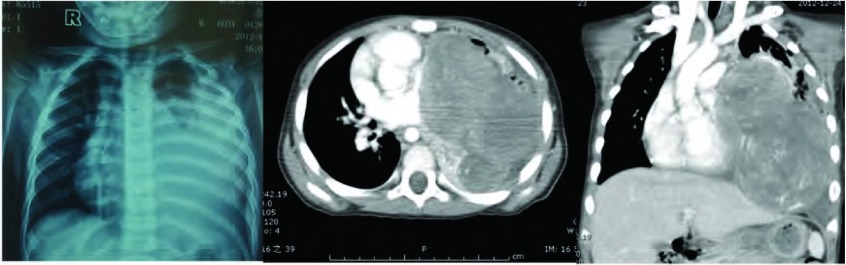
胸片及胸部CT增强示左侧胸腔有一大小约10 cm×10 cm×8 cm哑铃状分叶肿物，增强检查病灶可见不规则强化影。 Chest roenrtgenography and contrast enhanced CT scan of the chest: a giant dumb-bell sublobe mass (10 cm×10 cm×8 cm) in the left thoracic cavity. The solid portion of the mass shows a slight contrast enhancement.

**2:**
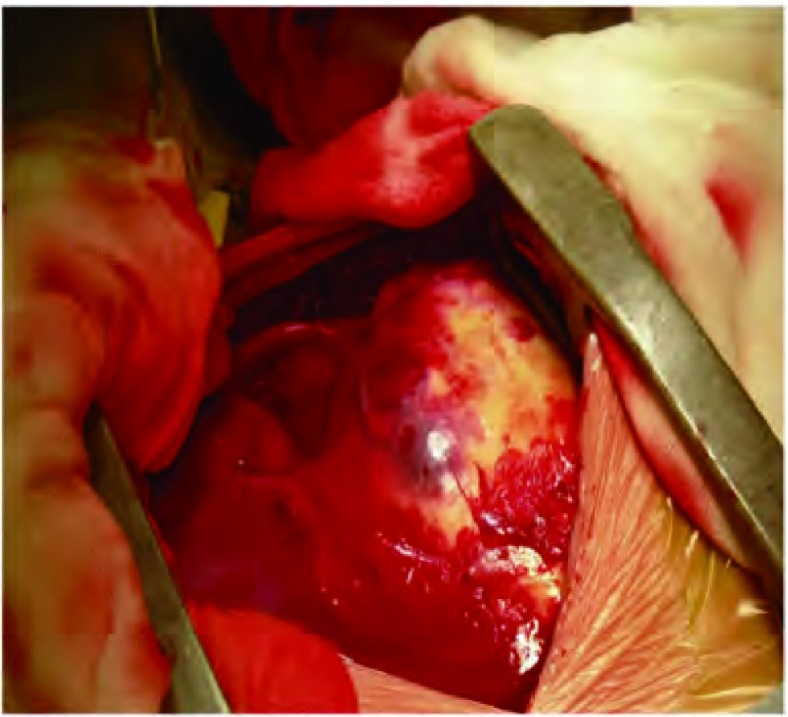
术中见胸腔巨大肿物压迫肺组织致肺不张 intraoperative view shows a giant mass compresses the lung to atelectasis

**3:**
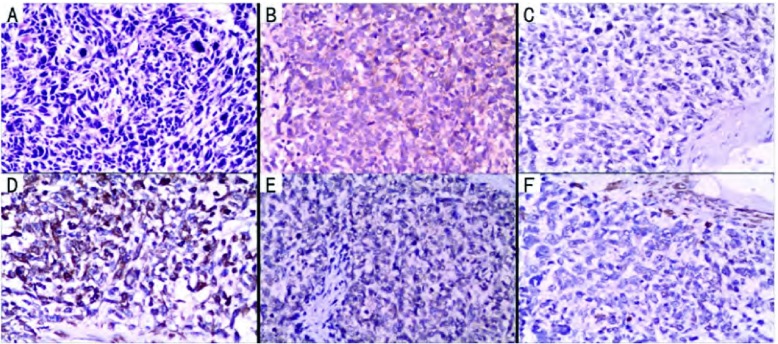
病理诊断：原始神经外胚层瘤。A：光镜下显示小圆细胞肿瘤（HE, ×200）；B：免疫组织化学检查示CD99（+）（EnVision, ×200）；C：免疫组织化学检查示NeuN（+）（EnVision, ×200）；D：免疫组织化学检查示Nestin（+）（EnVision, ×200）；E：免疫组织化学检查示CKp（+）（EnVision, ×200）；F：免疫组织化学检查示Vimentin（+）（EnVision, ×200）。 Pathologic diagnosis: primitive neuroectodermal tumor (PNET). A:Microphotograph is showing small round cell tumor (HE, ×200); B: Immunohistochemistry shows CD99(+)(EnVision, ×200); C: Immunohistochemistry shows positivity of tumor cells to NeuN (EnVision, ×200); D: Immunohistochemistry shows positivity of tumor cells to Nestin (EnVision, ×200); E: Immunohistochemistry shows positivity of tumor cells to CKp (EnVision, × 200); F:Immunohistochemistry shows positivity of tumor cells to Vimentin (EnVision, ×200).

**4:**
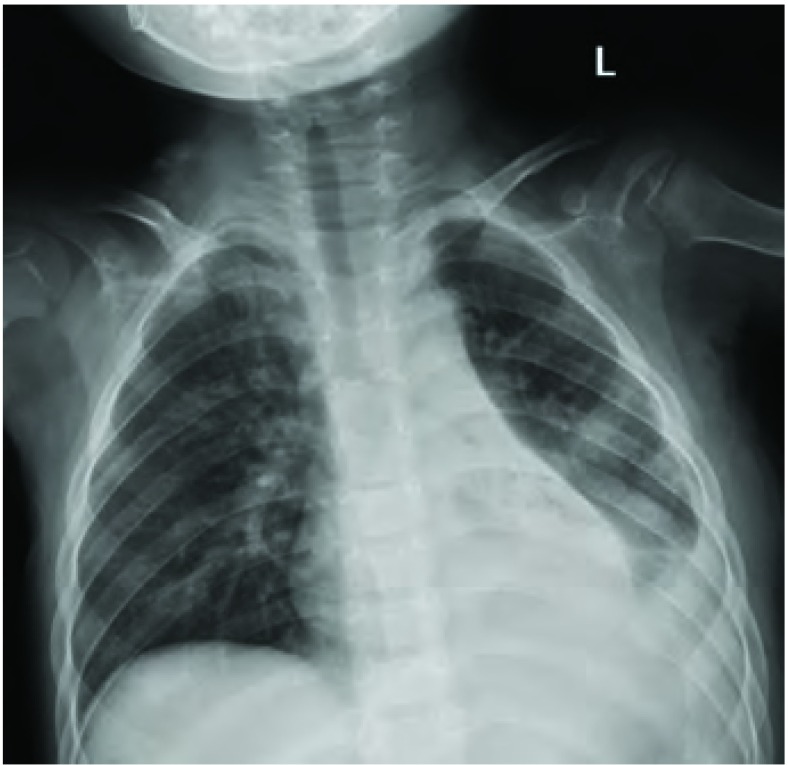
术后复查胸片示左肺复张良好 Postoperative chest roenrtgenography shows that the left lung recovered well

## 2 讨论

PNET是一种罕见的起源于原始神经管胚基细胞的未分化的高度恶性肿瘤。Stout^[1]^于1918年对其形态特征做了首次描述，Hart和Earle^[2]^于1973年首次提出PNET的概念。1993年WHO中枢神经系统肿瘤组织学分类中首次将PNET列入其中^[3]^。根据肿瘤发生来源及部位不同，PNET被分为中枢性PNET（cPNET）和外周性PNET（pPNET）。pPNET可发生于任何年龄，儿童和青少年多见；多见于躯干和四肢，尤其是椎旁区、胸壁、肢体和腹膜后，也见于实质脏器^[4]^。发生于纵隔的PNET较为罕见，容易误诊。近年国内报道的纵隔PNET仅见4例^[5-8]^，本例巨大纵隔PNET经手术完整切除，实为罕见。

胸部PNET临床表现以胸痛、胸闷气促和咳嗽三大症状为特征；其影像学表现缺乏特异性，术前诊断困难，易被误诊^[9]^。纵隔PNET影像学可表现为轮廓清楚、边缘光滑的巨大胸内软组织影，密度不均匀，常有液化区。增强CT扫描见肿物有不均匀性增强，有时与周围脏器或组织界限不清，侵犯胸膜或心包者出现胸腔积液或心包积液。本例患儿以胸痛为主要表现，纵隔肿物巨大，哑铃状分叶，压迫左侧上下叶肺致肺不张，术前诊断考虑为肺母细胞瘤、恶性畸胎瘤。影像学检查提示肿瘤有较完整包膜，术中亦发现肿瘤包膜较完整，但内部肿物鱼肉样，包膜与周围组织粘连紧密，提示为恶性肿瘤。

病理学上PNET表现为大小形态一致的小圆细胞，其诊断依靠特征性的镜下表现，但还需免疫组化来确诊。1991年Schmidt^[10]^提出了PNET诊断标准：至少表达两个不同的神经性标记和/或有Homer-Wright菊形团。CD99是PNET敏感而具有诊断价值的标记，其阳性率可达80%-95%。本例患儿CD99（+）、S100灶性（+）、NeuN（+）、Nestin（+）、Vimentin（+），根据细胞形态学表现及免疫组化结果诊断为PNET。PNET属于小圆细胞肿瘤，应与其它小圆细胞性肿瘤鉴别，主要有神经母细胞瘤、胚胎性横纹肌肉瘤、恶性淋巴瘤及小细胞未分化癌。鉴别方法主要是通过免疫组化。此外，t（11; 22）（q24; q12）染色体易位是PN ET分子生物学的染色体标记^[11]^，通过检测到其异位融合基因*EW/FLI-1*表达也是一种鉴别方法^[12]^。

PNET恶性程度高，预后极差。Ushigome等^[13]^报道23例患者只有2例存活8年，其余21例均于3个月-9个月内死亡。梁春梅等^[14]^报道了16例PNET，总生存期为3个月-67个月，中位生存期为26个月。PNET最有效的治疗措施是外科手术，对放化疗较为敏感，术后需化疗和放疗；化疗多采用蒽环类抗生素和烷化剂为基础的化疗方案^[15]^，但效果并不理想。PNET治疗失败主要原因是局部复发及远处转移。目前尚无应用靶向药物治疗的相关报道。本例患儿经手术完整切除病灶，术后已行两周期化疗，术后两个月复查胸部CT及腹部彩超，考虑局部复发及远处转移，提示预后不佳，仍需继续治疗观察。
